# White light-induced cell apoptosis by a conjugated polyelectrolyte through singlet oxygen generation†

**DOI:** 10.1039/c8ra00774h

**Published:** 2018-03-01

**Authors:** Jiamei Liang, Pan Wu, Chunyan Tan, Yuyang Jiang

**Affiliations:** Department of Chemistry, Tsinghua University Beijing 100084 P. R. China; The State Key Laboratory of Chemical Oncogenomics, The Graduate School at Shenzhen, Tsinghua University Shenzhen 518055 P. R. China tancy@sz.tsinghua.edu.cn +86-755-26036533; School of Pharmaceutical Sciences, Tsinghua University Beijing 100084 P. R. China

## Abstract

A cationic conjugated polyelectrolyte (CPE) PPET3 with a poly(*p*-phenylene ethynylene terthiophene) backbone and quaternary ammonium side chains was designed and synthesized. It serves as an efficient photosensitizer for photodynamic therapy under white light irradiation and induces cell death through the mitochondrial apoptosis pathway.

## Abstract 2

A conjugated polyelectrolyte PPET3 functions as an efficient photosensitizer for photodynamic therapy and induces cell apoptosis under white light irradiation.

## Graphical abstract

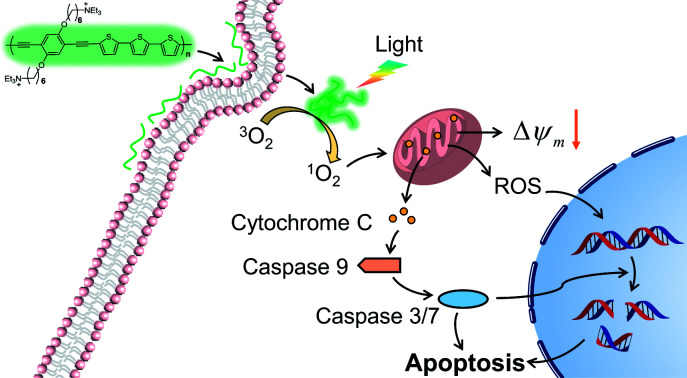

Reactive oxygen species (ROS) act as a double-edged sword in cells in which moderate levels of ROS function as important messengers in intracellular signalling pathways.^1–3^ However, overproduction of ROS may disrupt cellular homeostasis, cause oxidative damage to cellular constituents, and result in cell growth arrest or cell death.^2,4^ Thus, it is a promising strategy to utilize ROS as cytotoxic agents to induce cell apoptosis. Photodynamic therapy (PDT) is an attractive ROS-mediated therapeutic modality.^5^ It utilizes the combination of a photosensitizer (PS), light (usually in the visible spectrum), and oxygen molecules to produce excess intracellular ROS, predominantly highly reactive singlet oxygen (^1^O_2_) that can oxidize and damage biomolecules.^6,7^ However, the cell death mechanism induced by PDT is complex and depends on multiple factors, such as intracellular localization of PS, PS concentration, and light dose.^8^ Therefore, the mechanism of cell death is an ongoing topic of investigation in PDT.

Various chemical compounds have been investigated as PSs in PDT, including porphyrins, phenothiazines, cyanines, borondipyrromethene dyes, and transition metal complexes.^9–11^ In recent years, conjugated polyelectrolytes (CPEs), characterized by a delocalized π-electronic backbone and ionic side chains, have aroused considerable attention as admirable PSs due to their interesting optical properties, such as strong light-harvesting capability, high fluorescence quantum yields, and good photostability.^12–14^ Since a cationic poly(*p*-phenylene ethynylene) (PPE) was first reported to kill bacteria under visible light irradiation in 2005,^15^ a variety of CPEs with backbones of PPEs,^16,17^ poly(phenylene vinylene)s (PPVs),^18^ poly(fluorine-*co*-phenylene)s (PFPs),^19^ and poly(thiophene)s (PTs)^20,21^ have been developed for use in light-activated antimicroorganism therapy. Moreover, other researches that have gained much attention include anticancer systems based on CPEs and the combination of CPEs with other traditional PSs to enhance ^1^O_2_ generation,^22–25^ for example, the combined use of CPEs with porphyrin to enhance the generation of ^1^O_2_. However, few studies exist on the cellular response to CPE-mediated PDT. Even though a cationic PT has been reported to induce cell apoptosis by increasing activation of caspase-3 under irradiation,^22^ the upstream and downstream signal events elicited by CPEs-mediated PDT are still not fully understood.

In this work, we report a cationic poly(*p*-phenylene ethynylene terthiophene) (PPET3) as a sensitizer for effective PDT under white light irradiation. Compared with the reference polyelectrolyte PPE, PPET3 showed more efficient photo-induced ^1^O_2_ generation and higher photocytotoxicity under identical conditions. The mechanisms of cell apoptosis induced by PPET3 in PDT treatment were evaluated through flow cytometric analysis, mitochondrial membrane potential (MMP) measurement, western blot, and confocal imaging analysis. The results suggest that PPET3 can induce mitochondrial apoptosis upon white light irradiation (Scheme 1).

**Scheme 1:**
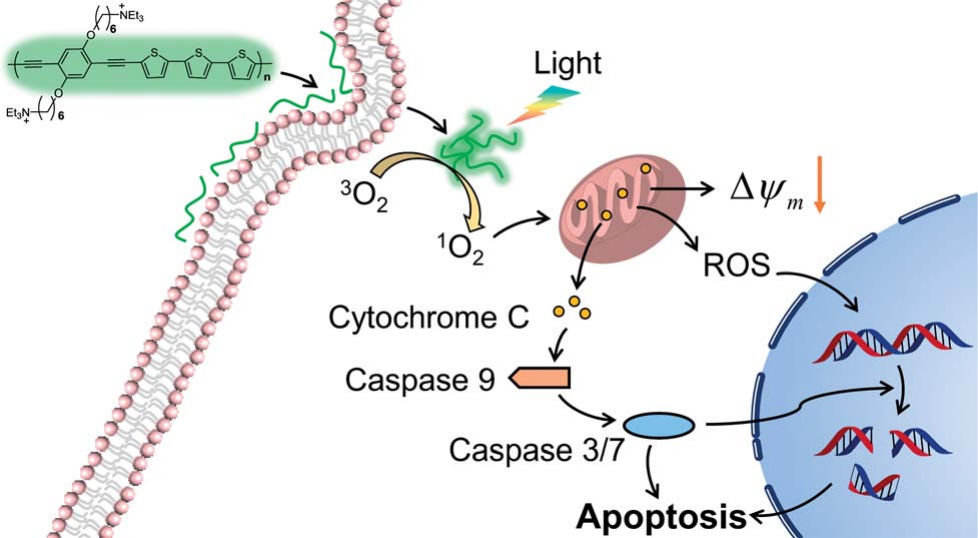
Illustration of PPET3 as a photosensitizer to induce cell apoptosis.

The molecular structures of PPET3 and PPE are shown in the ESI (Scheme S1†), and their photophysical properties were characterized in pure water. PPET3 exhibited a wide absorption band located at 350–600 nm with a peak at 450 nm (Fig. S1, ESI†), covering the spectral regions ranging from violet, blue, green to yellow and suggesting that the polymer can be excited by broad spectrum white light. However, PPE showed a narrower absorption band in the wavelength range of 350–500 nm (absorption peak at 393 nm), which might limit its excitation efficiency under white light irradiation. In addition, both molar extinction coefficients of PPET3 and PPE were determined to be about 1.5 × 10^4^ M^−1^ cm^−1^, indicating that the two CPEs have strong light-harvesting capability.

Since PDT efficacy mostly depends on the ^1^O_2_ generation of the photosensitizer, the ability of PPET3 and PPE to photosensitize ^1^O_2_ was evaluated using 9,10-anthracenediyl-bis(methylene) dimalonic acid (ABDA) as the ^1^O_2_ detection reagent. ABDA can be selectively oxidized by ^1^O_2_ to form its corresponding endoperoxide component (Fig. 1a), which exhibited photobleaching.^26^ Therefore, the loss of absorbance of ABDA can quantify the amount of ^1^O_2_ generation in solution. Fig. 1b shows the absorption spectra of ABDA in aqueous solutions containing different concentrations of PPET3 (5, 10, 20, and 50 μM) as a function of exposure time of white light (400–800 nm, power: 1 W, fluence rate: 100 mW cm^−2^). Under irradiation, the absorption peaks of ABDA monotonically decreased in intensity with an increase in exposure time, indicating an increased yield of ^1^O_2_. Similar behaviour was observed in the case of ABDA in the presence of PPE under identical experimental conditions (Fig. S2a, ESI†). The ratio of the characteristic absorption peak of ABDA at 378 nm before and after irradiation against exposure time is summarized (Fig. 1c and S2b, ESI†). In the presence of 5, 10, 20, and 50 μM PPET3 and after 10 min of white light illumination, the relative absorbance of ABDA at 378 nm decreased to 74.6%, 61.4%, 36.0%, and 12.3%, respectively, and decreased to 84.7%, 75.9%, 65.1% and 30.0% for the respective concentrations of PPE. The decrease rate of absorbance intensity in the presence of PPET3 was faster than that in PPE with equal concentration. That is, PPET3 displayed better ^1^O_2_ generation ability, which was attributed to the intersystem crossing effect enhanced by terthiophene units.^27,28^ There was no obvious decrease in absorbance for the solution containing ABDA without any CPEs after irradiation, confirming that the decrease in ABDA absorbance intensity was caused by ^1^O_2_ generated from photosensitive CPEs instead of white light illumination.

**Fig. 1:**
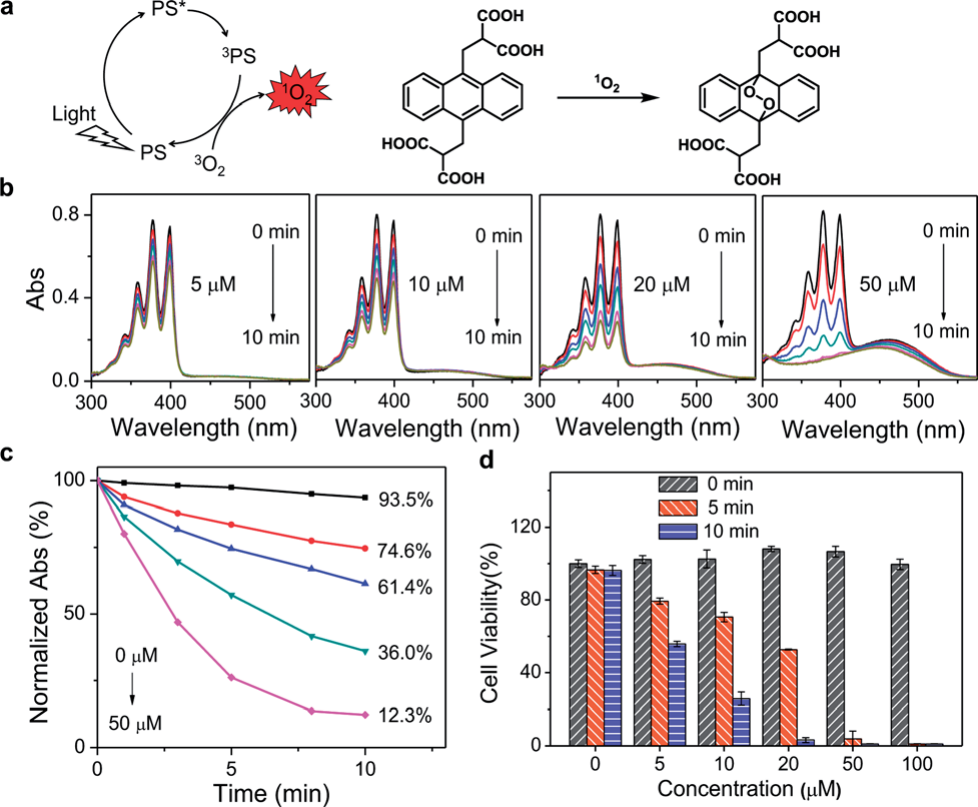
(a) Schematic diagram of ^1^O_2_ generation and chemical reaction of ABDA with ^1^O_2_. (b) Absorption spectral changes of ABDA in the presence of 5, 10, 20, and 50 μM PPET3 over different periods of exposure time. (c) Plot of normalized absorbance of ABDA at 378 nm against exposure time in the solutions containing various concentrations of PPET3. (d) Relative cell viability of HeLa cells incubated with PPET3 at a series of concentrations with or without white light irradiation.

To verify the PDT efficacy of PPET3 and PPE, the dark-toxicity and phototoxicity against human cervical carcinoma (HeLa) cells were measured using a 3-(4,5-dimethylthiazol-2-yl)-2,5-diphenyltetrazolium bromide (MTT) assay. We first explored the parameters of the PDT dose (PS concentration and irradiation time). HeLa cells were treated with graded concentrations of PPET3 and PPE (5, 10, 20, 50, and 100 μM) and different doses of white light (5 and 10 min). As illustrated in Fig. 1d, the cell viability decreased rapidly with increasing concentrations of PPET3 and increasing white light dose. In contrast, PPET3 showed no obvious cytotoxicity (cell viability > 99%) in the dark even up to a concentration of 100 μM, suggesting water-soluble PPET3 has excellent biocompatibility and low dark cytotoxicity. In addition, the cells maintained high viability when white light irradiation was performed alone. These results demonstrate that photocytotoxicity was induced by ^1^O_2_ generated from photosensitive PPET3 and not by the irradiating white light. The reference polyelectrolyte, PPE generated less ^1^O_2_ under white light irradiation than PPET3 in identical concentration and displayed much less photocytotoxicity against HeLa cells under similar experimental conditions (Fig. S3, ESI†). Even at the highest tested concentration (100 μM), HeLa cells maintained 84% viability after exposure to white light for 10 min. We speculate that the PDT efficacy of a photosensitizer in HeLa cells depends both on its ^1^O_2_ generation efficiency and other parameters, such as cellular uptake and retention.

Earlier studies found that the internalized PPET3 translocated from lysosomes to mitochondria upon white light irradiation.^29^ However, the responses of HeLa cells to the excessive ^1^O_2_ have not yet been investigated. Since mitochondria plays a vital role in cell apoptotic pathway, we explored the capability of ^1^O_2_ to induce cell apoptosis. The effects of PPET3-mediated PDT on apoptotic death in Annexin V-mFluor Violet 450/PI staining HeLa cells were assessed by flow cytometric analysis. Fig. 2 shows the percentages of viable (Annexin V negative, PI negative), early apoptotic (Annexin V positive, PI negative), late apoptotic (Annexin V positive, PI positive), and necrotic (Annexin V negative, PI positive) cells after different treatments. The cells treated with PPET3 (5 and 10 μM) had minimal detrimental effects without white light irradiation. However, after PPET3-treated cells were exposed to white light, the cells exhibited an increased percentage of both early apoptotic cells and late apoptotic cells. For 5 μM PPET3-treated cells under white light, the early apoptotic cells were 4.56% after 5 min irradiation, and increased to 11.6% after 10 min irradiation. When the treated concentration of PPET3 was increased to 10 μM, the percentage of early apoptotic cells was slightly increased to 9.32% and 12.4% after 5 and 10 min of irradiation, respectively. These results serve as evidence that PPET3-mediated PDT is capable of inducing cell apoptosis.

**Fig. 2:**
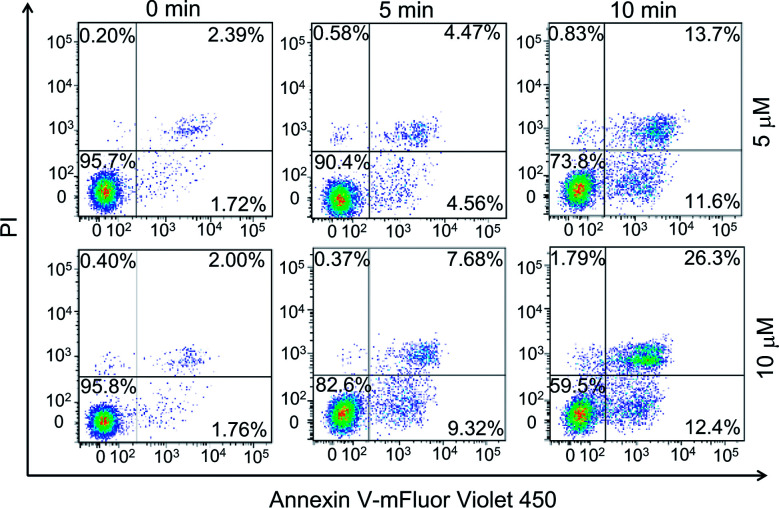
Flow cytometry quantification of different treatments of HeLa cells labeled with Annexin V-mFluor Violet 450/PI. The cells were incubated with PPET3 (5 and 10 μM) and irradiated by white light for 5 or 10 min.

Since early apoptotic cells undergo characteristic depolarization of mitochondria,^30^ detection of the mitochondrial membrane potential (MMP, Δ*Ψ*_m_) using tetramethylrhodamine (TMRM) was carried out by flow cytometry. TMRM is a lipophilic cationic rhodamine derivative, which will accumulate within mitochondria in an inverse proportion to Δ*Ψ*_m_.^31^Fig. 3 displays the histogram plots of HeLa cells after the treatment of PPET3 alone or combined with PPET3 and white light irradiation. Compared to HeLa cells treated solely with PPET3, the cells treated by combined PPET3 and irradiation exhibited weaker TMRM fluorescence, indicating mitochondrial membrane disruption in HeLa cells after combined treatment. Moreover, the intensity of TMRM fluorescence weakened further when the irradiation time was prolonged from 5 to 10 min. The above studies indicate that the MMP decreased with an increase in PPET3 concentration or light dose, which agree with the results from Annexin V-mFluor staining in Fig. 2.

**Fig. 3:**
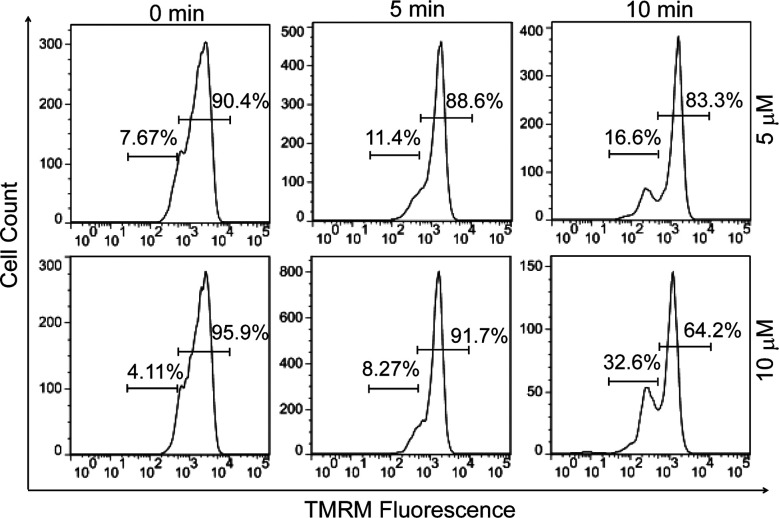
Flow cytometry quantification of different treatments of HeLa cells labeled with TMRM (200 nM). The cells were incubated with PPET3 (5 and 10 μM) and irradiated with white light for 5 or 10 min.

The loss of MMP was reported to facilitate cytochrome C release and activate apoptotic cascade reaction.^32^ To further analyse the mitochondria-mediated apoptosis pathway activated by PPET3-mediated PDT, the expression of related proteins was tested using western blotting. HeLa cells treated with PPET3 alone and with white light irradiation alone had nearly no changes in the expressions of the detected proteins compared with control HeLa cells (Fig. 4a, left). However, when the cells were treated with the combination of PPET3 and white light illumination, the expression of cleaved caspase-9, -3, and -7 increased, accompanying the cleavage of poly(ADP-ribose) polymerase (PARP), one of the main targets of cleaved caspase-3. Caspase-9 plays a pivotal role in the intrinsic mitochondrial apoptosis pathway, and the cleavage of caspase-9 presumably triggers a cascade of caspase activation events,^33^ including the cleavage of caspase-7 and -3. PARP activated by cleaved caspase-3 is essential for cell apoptosis.^34^ Our results reveal that PPET3 can efficiently induce apoptosis of HeLa cells *via* the mitochondrial apoptotic pathway.

**Fig. 4:**
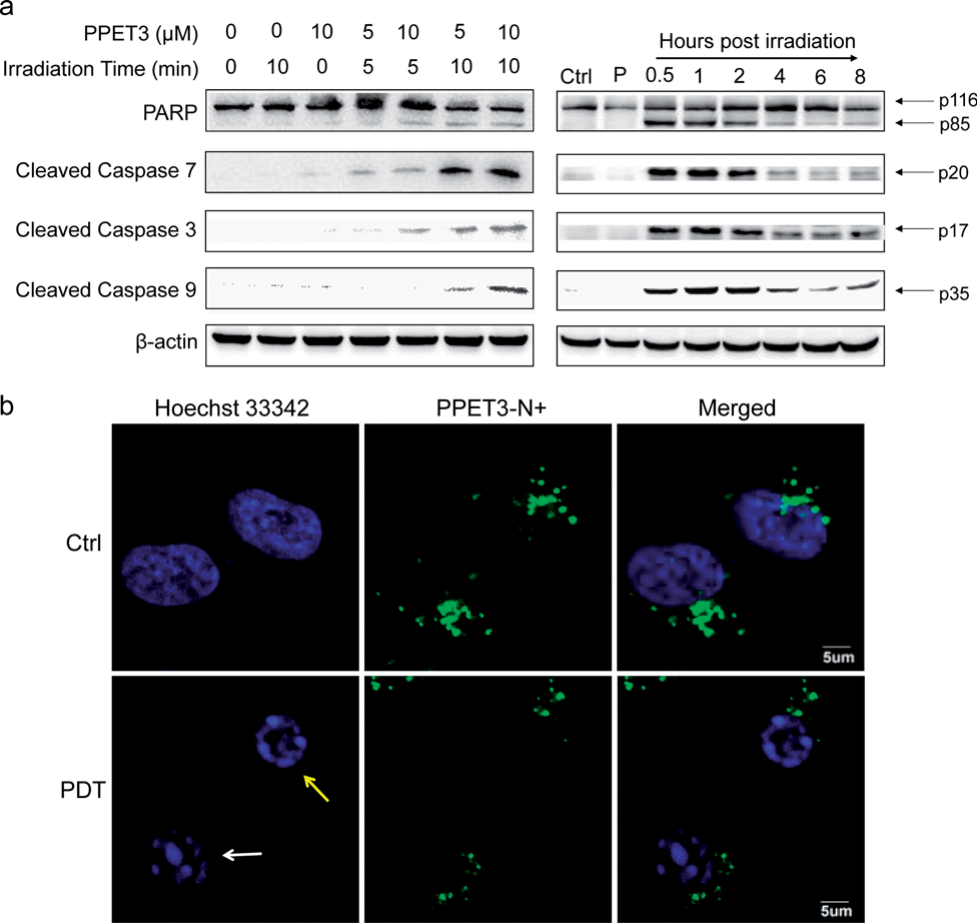
(a) Western blot analysis of the expression of apoptosis-related proteins in HeLa cells. Left: HeLa cells were incubated with PPET3 (5 and 10 μM) and irradiated by white light for 5 or 10 min. The expression of the proteins was evaluated at 8 h post-treatment. Right: HeLa cells were incubated with PPET3 (5 μM) and irradiated by white light for 10 min. The expression of the proteins was evaluated at 0.5, 1, 2, 4, 6, and 8 h post-treatment, respectively. Ctrl: untreated cells, P: cells incubated with 5 μM PPET3 alone. (b) Confocal fluorescence images of PPET3 (5 μM)-treated HeLa cells after white light irradiation for 10 min. Cells were stained with Hoechst 33342. Scale bar: 5 μm.

Furthermore, time-dependent cell apoptosis was also investigated using western blotting. The cells were treated with a combination of 5 μM PPET3 and 10 min of white light illumination. The expression of the caspase family as well as PARP was evaluated at 0.5, 1, 2, 4, 6, and 8 h post-treatment (Fig. 4a, right). In cells fractionated within 0.5–2 h post-treatment, there was almost no considerable change in the expression of cleaved caspase-9, -3, and -7 as well as cleaved PARP. However, when time was extended to 4 and 6 h, a decreasing amount of the cleaved caspases and PARP was observed. In addition, levels of the proteins were found to increase slightly 8 h post-treatment.

To explore the morphological changes of nucleus induced by PPET3-mediated PDT treatment, Hoechst 33342 which has a bright blue fluorescence when combined with DNA double strands, was employed to label the nuclei. As shown in Fig. 4b, the nucleus of control cells treated with 5 μM PPET3 alone were evenly stained by Hoechst 33342. In contrast, the cells treated with the combination of 5 μM PPET3 and 10 min of white light illumination displayed chromatin margination (the yellow arrow in Fig. 4b), the disassembly of chromosomal territory, and formation of spatially organized nuclear apoptotic bodies (the white arrow in Fig. 4b). These results further indicate that the cells underwent apoptotic cell death after PPET3-mediated PDT treatment.

## Conclusions

In conclusion, we describe the utilization of PPET3 containing terthiophene units in its backbone as a PDT agent for HeLa cells. PPET3 exhibited good biocompatibility, low cytotoxicity in dark, and a high yield of ^1^O_2_ under white light irradiation. The photocytotoxicity of PPET3 mostly depended on the concentration of the sensitizer and white light dose. The combined treatment of PPET3 with white light irradiation efficiently induced mitochondrial depolarization and cell apoptosis. Additionally, the activation of caspase-9, -3, and -7 as well as PARP was observed by western blotting, further confirming the activation of mitochondrial apoptosis. The successful new application of PPET3 in PDT and its apoptosis-inducing property under white light irradiation demonstrates its potential as a promising photosensitizer in disease therapeutics.

## Methods

### Determination of singlet oxygen generation

A chemical oxidation method based on ABDA was used to monitor the ^1^O_2_ generation from photosensitive PPET3 and PPE. ABDA is a water soluble derivative of anthracene that can be converted to its corresponding endoperoxide after specifically oxidized by ^1^O_2_, which results in a decrease in its absorbance at 378 nm. A white light LED lamp (wavelengths ranging from 400 to 800 nm, power: 1 W, 100 mW cm^−2^) was employed as the light source. The solutions containing various concentrations of PPET3 or PPE (5, 10, 20 and 50 μM) and ABDA/DMSO were homogeneously mixed in a 96-well plate, reacted in the dark for 15 min and then irradiated with white light for different time durations (0, 1, 3, 5, 8, 10, 12 and 15 min). The control experiment was carried out with ABDA under the same irradiation condition but without any polyelectrolytes. The absorption spectra of ABDA was recorded with a plate reader at designated time intervals.

### Cell viability assay

The viability of HeLa cells exposed to PPET3 or PPE were evaluated *via* MTT assay. HeLa cells were seeded onto 96-well plates (10 000 cells per well) and incubated for 24 h. Then the culture medium was replaced with fresh medium containing PPET3 or PPE at various concentrations (0, 5, 10, 20, 50 and 100 μM). After 2 h of incubation, HeLa cells were washed twice with PBS buffer to ensure the extracellular conjugated polyelectrolytes was completely removed and then incubated with fresh medium for another 10 h to completely internalize the polyelectrolytes. The cells were exposed to white light for 5 or 10 min to induce phototoxicity or kept in dark as control. After another 24 h cell culture, 10 μL of MTT (0.5 mg mL^−1^) was added into each well. The supernatant liquid was carefully removed after 4 h of incubation, then 100 μL of DMSO was added into each well to dissolve the formazan crystals produced by the live cells. The optical density (OD) at 570 nm was read with a plate reader.

### Flow cytometry analysis of apoptosis and necrosis cells

HeLa cells were seeded in a 12-well plate (200 000 cells per well) and incubated for 24 h. Cells were treated with PPET3 (5 or 10 μM) in medium for 2 h and washed twice to remove the remaining polyelectrolytes. After additional 10 h of culture, the cells were irradiated with white light for various lengths of time (0, 5 and 10 min). Every two wells of cells were treated in the same condition and harvested into a centrifuge tube at 2 h post-treatment. The cells were washed twice with pre-cooling PBS, resuspended in 200 μL of assay buffer and stained with 2 μL of Annexin V-mFluor Violet 450 (*E*_x_: 405 nm, *E*_m_: 450 nm) and 2 μL of propidium iodide (PI) at room temperature for 30 min in the dark. After added 300 μL of assay buffer, the samples were analyzed on a flow cytometer within 1 h.

### Measurement of mitochondrial membrane potential (MMP) with TMRM

HeLa cells were treated and harvested as described in the flow cytometry analysis of apoptosis and necrosis cells. The samples were washed twice with pre-cooling PBS and incubated with 200 nM TMRM (*E*_x_: 549 nm, *E*_m_: 573 nm) at 37 °C for 30 min in the dark. After washed twice with pre-cooling PBS, the cells were analyzed with a flow cytometer within 1 h. Results were expressed as the proportion of cells with low TMRM fluorescence indicating the loss of MMP.

### Western blot analysis of the activation of caspases and PARP

HeLa cells were seeded in a 12-well plate and incubated for 24 h. Then, cells were treated with PPET3 (5 μM and 10 μM) in culture medium for 2 h and washed twice to remove untaken samples. After additional 10 h of culture, each well were irradiated with white light for 5 min or 10 min. The cells were resuspended, washed and collected at 8 h post-treatment. After lysed with RIPA lysis buffer and PMSF, the samples were centrifuged and the supernatant was collected. To ensure an equal amount of protein was loaded, the protein concentrations of the samples were measured with BCA protein assay. Cell lysate was mixed with sample loading buffer and then heated to 100 °C for 10 minutes. Equal amounts of denatured proteins (30 μg) were loaded into a 12% SDS-PAGE gel and gel electrophoresis was performed under 100 V for 2 h. The proteins were detected by western blot.

To investigate the kinetics of caspase family and PARP cleavage, HeLa cells were seeded in a 12-well plate and incubated for 24 h. Then, cells were treated PPET3 (5 μM) in culture medium for 2 h and washed twice to remove untaken samples. After additional 10 h of culture, each well were treated with white light for 10 min. The cells were resuspended, washed and collected at various intervals after PPET3-induced PDT. The remaining steps were as previous describing.

### PDT-induced apoptosis with Hoechst 33342 staining fluorescence imaging

HeLa cells were seeded on 22 mm glass coverslips coated with poly(l-lysine) and the coverslips were placed at the bottom of 35 mm culture dishes. After incubated for 24 h, the cells were treated with PPET3 (5 μM) in culture medium for 2 h, washed three times with PBS and incubated for 10 h. The cells were exposed to white light for 10 min or kept in dark as control. At 8 h post-treatment, the cells were stained with 800 μL of assay buffer containing 5 μL of Hoechst 33342 in the dark at 4 °C for 20 min, washed twice with PBS, fixed in 4% paraformaldehyde for 10 min at room temperature and washed three times with PBS. The coverslips were sealed and fluorescence images were taken by confocal microscopy and fluorescent microscopy.

## Conflicts of interest

There are no conflicts to declare.

## Supplementary Material

RA-008-C8RA00774H-s001
