# Supplementary material for: White light-induced cell apoptosis by a conjugated polyelectrolyte through singlet oxygen generation
Source: RSC Adv. 2018 Mar 1;8(17):9218–22. doi: 10.1039/c8ra00774h (PMC9078657; doi:10.1039/c8ra00774h)
Supplement: RA-008-C8RA00774H-s001 [file RA-008-C8RA00774H-s001.pdf]

Electronic Supplementary Material (ESI) for RSC Advances.
This journal is © The Royal Society of Chemistry 2018

Supplementary Information

White light-induced cell apoptosis by a conjugated polyelectrolyte through singlet oxygen generation

Jiamei Liang,[ab] Pan Wu,[ab] Chunyan Tan,*[ab] and Yuyang Jiang[bc]

[a] Department of Chemistry, Tsinghua University, Beijing 100084, P. R. China.

[b] The State Key Laboratory of Chemical Oncogenomics, the Graduate School at Shenzhen, Tsinghua University, Shenzhen 518055, P. R. China. E-mail: tancy@sz.tsinghua.edu.cn; Tel: +86-755-26036533.

[c] School of Pharmaceutical Sciences, Tsinghua University, Beijing 100084, P. R. China.

Experimental

Materials. Conjugated polyelectrolytes PPET3 was synthesized by Pd-catalysed Sonogashira coupling reaction according to the literature procedure[1] with stock aqueous solution concentration of 346 μM, and PPE with stock solution concentration of 2 mM was used as the reference CPE (the concentrations are provided as polymer repeat unit concentration, [PRU]). Fetal bovine serum (FBS) was purchased from Gibco (New York, America). Minimum essential medium eagle (MEM) was purchased from Corning cellgro (Virginia, America). Phosphate-buffered saline (PBS), trypsin-EDTA solution, 3-(4,5-dimethylthiazol-2-yl)-2,5-diphenyltetrazolium bromide (MTT), Cytochrome c antibody, β-actin antibody, Hoechst 33342, and BeyoECL star chemiluminescence kit were purchased from Beyotime Biotech Co. (Shanghai, China). 9, 10-anthracenediyl-bis(methylene) dimalonic acid (ABDA) was purchased ftrom Sigma-Aldrich Co. LLC. (Shanghai, China). Annexin V binding apoptosis assay kit was purchased from AAT Bioquest Inc. (California, America). [Tetramethylrhodamine, methyl ester, perchlorate] (TMRM) was purchased from Absin Bioscience Inc. (Shanghai, China). PARP antibody, cleaved caspase-3 antibody, cleaved caspase-7 antibody, and cleaved caspase-9 antibody were purchased from Cell Signaling Technology Inc. (Massachusetts, America).

Determination of singlet oxygen generation. A chemical oxidation method based on ABDA was used to monitor the $^1O_2$ generation from photosensitive PPET3 and PPE.

ABDA is a water soluble derivative of anthracene that can be converted to its corresponding endoperoxide after specifically oxidized by $^1O_2$, which results in a decrease in its absorbance at 378 nm. A white light LED lamp (wavelengths ranging from 400 to 800 nm, power: 1 W, 100 mW cm^{-2}) was employed as the light source. The solutions containing various concentrations of PPET3 or PPE (5, 10, 20 and 50 μM) and ABDA/DMSO were homogeneously mixed in a 96-well plate, reacted in the dark for 15 min and then irradiated with white light for different time durations (0, 1, 3, 5, 8, 10, 12 and 15 min). The control experiment was carried out with ABDA under the same irradiation condition but without any polyelectrolytes. The absorption spectra of ABDA was recorded with a Tecan Infinite M1000 Pro plate reader at designated time intervals.

Cell Culture. Human cervical carcinoma cells (Hela cells) were used as a model cell line and cultured in MEM medium supplemented with 10% FBS at 37°C with 5% CO_2 in a humidified incubator. Cells in the exponential phase of growth were used in the experiments.

Cell viability assay. The viability of Hela cells exposed to PPET3 or PPE were evaluated via MTT assay. Hela cells were seeded onto 96-well plates (10,000 cells/well) and incubated for 24 h. Then the culture medium was replaced with fresh medium containing PPET3 or PPE at various concentrations (0, 5, 10, 20, 50 and 100 μM). After 2 h of incubation, Hela cells were washed twice with PBS buffer to ensure the extracellular conjugated polyelectrolytes was completely removed and then incubated

with fresh medium for another 10 h to completely internalize the polyelectrolytes. The cells were exposed to white light (400-800 nm, power: 1W, 100 mW cm^{-2}, each well for 5 or 10 min) to induce phototoxicity or kept in dark as control. After another 24 h cell culture, 10 μL of MTT (0.5 mg/mL) was added into each well. The supernatant liquid was carefully removed after 4 h of incubation, then 100 μL of DMSO was added into each well to dissolve the formazan crystals produced by the live cells. The optical density (OD) at 570 nm was read with a Tecan Infinite M1000 Pro plate reader. The cell viability was calculated using the following equation:

$$\text{cell viability} = \left(\frac{OD_{sample} - OD_{blank}}{OD_{control} - OD_{blank}}\right) \times 100\% .$$

Flow cytometry analysis of apoptosis and necrosis cells. Hela cells were seeded in a 12-well plate (200,000 cells/well) and incubated for 24 h. Cells were treated with PPET3 (5 or 10 μM) in medium for 2 h and washed twice to remove the remaining polyelectrolytes. After additional 10 h of culture, the cells were irradiated with white light (400-800 nm, power: 1W, 100 mW cm^{-2}) for various lengths of time (0, 5 and 10 min). Every two wells of cells were treated in the same condition and harvested into a centrifuge tube at 2 h post-treatment. The cells were washed twice with pre-cooling PBS, resuspended in 200 μL of Assay Buffer and stained with 2 μL of Annexin V-mFluor Violet 450 (E_x: 405 nm, E_m: 450 nm) and 2 μL of propidium iodide (PI) at room temperature for 30 min in the dark. After added 300 μL of Assay Buffer, the samples were analyzed on the Becton Dickinson FACS Canto II flow cytometer within 1 h.

Measurement of mitochondrial membrane potential (MMP) with TMRM. Hela cells were seeded in a 12-well plate (200,000 cells/well) and incubated for 24 h. Cells were treated with the medium containing PPET3 (5 or 10 μM) for 2 h and washed twice with PBS. After incubated for 10 h, the cells were irradiated with white light (400-800 nm, power: 1W, 100 mW cm^{-2}) for different lengths of time (0, 5 and 10 min). Every two wells of cells were treated under the same condition and harvested as a sample at 2 h post-treatment. The samples were washed twice with pre-cooling PBS and incubated with 200 nM TMRM (E_x: 549 nm, E_m: 573 nm) at 37°C for 30 min in the dark. After washed twice with pre-cooling PBS, the cells were analyzed with the Becton Dickinson FACS Canto II flow cytometer within 1 h. Results were expressed as the proportion of cells with low TMRM fluorescence indicating the loss of MMP.

Western blot analysis of the activation of caspases and PARP. Hela cells were seeded in a 12-well plate (Thermo Scientific) and incubated for 24 h. Then, cells were treated with PPET3 (5 μM and 10 μM) in culture medium for 2 h and washed twice to remove untaken samples. After additional 10 h of culture, each well were irradiated with white light for 5 min or 10 min. The cells were resuspended, washed and collected at 8 h post-treatment. After lysed with RIPA lysis buffer and PMSF, the samples were centrifuged (18000× g, 10 min, 4°C) and the supernatant was collected. To ensure an equal amount of protein was loaded, the protein concentrations of the samples were measured with BCA protein assay. Cell lysate was mixed with 5× SDS-PAGE sample

loading buffer and then heated to 100°C for 10 minutes. Equal amounts of denatured proteins (30 μg) were loaded into a 12% SDS-PAGE gel and gel electrophoresis was performed using 1× Tris-glycine SDS Running Buffer under 100 V for 2 h. Proteins were transferred to PDVF membranes and the membranes were blocked with blocking buffer (1× TBST with 5% nonfat milk) for 1 h at room temperature. After washed three times for 5 min with 1× TBST, the membranes were incubated with diluted primary antibody in 1× TBST (1:1000) with gentle agitation at 4°C overnight. After incubation, the primary antibody was removed and membranes was washed three times for 5 min with 1× TBST. The membranes were incubated with secondary antibody in 1X TBST (1:2000) with gentle agitation for 2 h at room temperature. After incubation, the secondary antibody was removed and membranes was washed three times for 5 min with 1× TBST and developed using high sensitivity BeyoECL Star chemiluminescence kit. The membranes were visualized using a Tanon 5200 Multi Chemiluminescent imaging system.

To investigate the kinetics of caspase family and PARP cleavage, Hela cells were seeded in a 12-well plate (Thermo Scientific) and incubated for 24 h. Then, cells were treated PPET3 (5 μM) in culture medium for 2 h and washed twice to remove untaken samples. After additional 10 h of culture, each well were treated with white light for 10 min. The cells were resuspended, washed and collected at various intervals after PPET3-induced PDT. The remaining steps were as previous describing.

PDT-induced apoptosis with Hoechst 33342 staining fluorescence imaging. Hela cells were seeded on 22 mm glass coverslips coated with poly(L-lysine) and the coverslips were placed at the bottom of 35 mm culture dishes. After incubated for 24 h, the cells were treated with PPET3 (5 μM) in culture medium for 2 h, washed three times with PBS and incubated for 10 h. The cells were exposed to white light (400-800 nm, power: 1W, 100 mW cm^{-2}) for 10 min or kept in dark as control. At 8 h post-treatment, the cells were stained with 800 μL of assay buffer containing 5 μL of Hoechst 33342 in the dark at 4°C for 20 min, washed twice with PBS, fixed in 4% paraformaldehyde for 10 min at room temperature and washed three times with PBS. The coverslips were sealed and fluorescence images were taken by confocal microscopy (Olympus FV1000-IX81) and fluorescent microscopy (Olympus IX51).

Results

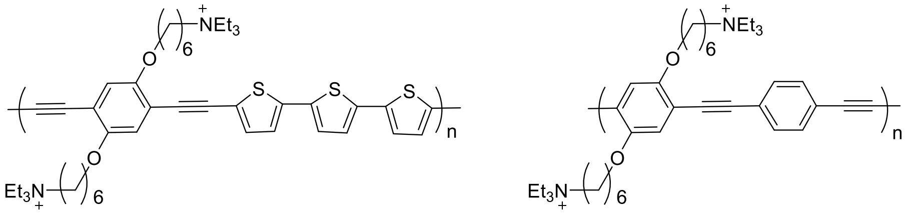

PPET3 **PPE**

Scheme S1. Chemical structures of PPET3 and PPE.

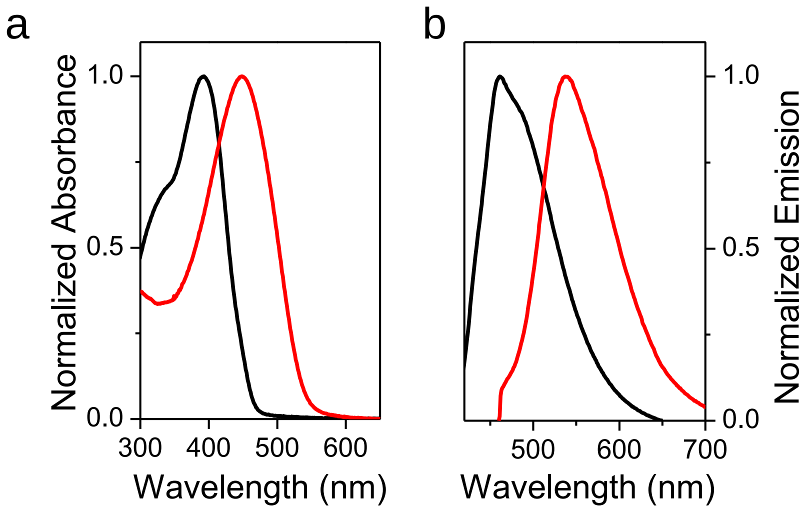

c

Polymer	$\lambda_{abs,\ max}$ (nm)	$\lambda_{em,\ max}$ (nm)	ε_{max} (10^4 $M^{-1}\cdot cm^{-1}$)
**PPET3**	450	537	1.8
**PPE**	393	461	1.3

Fig. S1 Photophysical features of PPET3 and PPE. (a) Normalized UV-visible absorption and (b) fluorescence emission spectra of PPET3 (red line) and PPE (black line) in aqueous solution. (c) Photophysical data of PPET3 and PPE. PPET3 and PPE were excited at 440 nm and 390 nm, respectively.

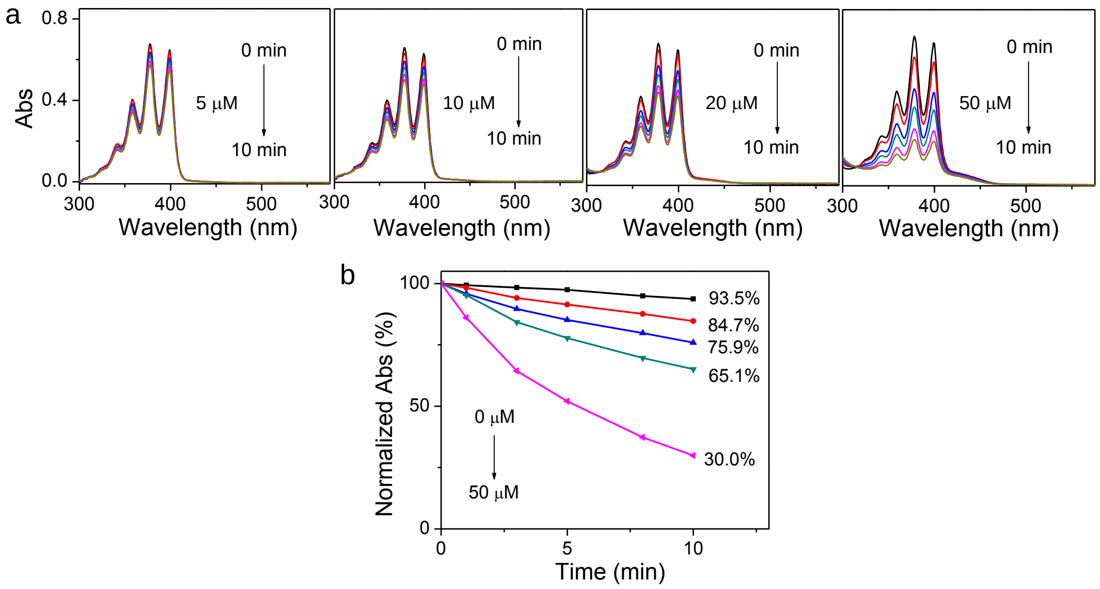

Fig. S2 Singlet oxygen generation from PPE under white light irradiation. (a) Absorption spectral changes of ABDA in the presence of 5, 10, 20, and 50 μM PPE over different periods of exposure time. (b) Plot of normalized absorbance of ABDA at 378 nm against exposure time in the solutions containing various concentrations of PPE.

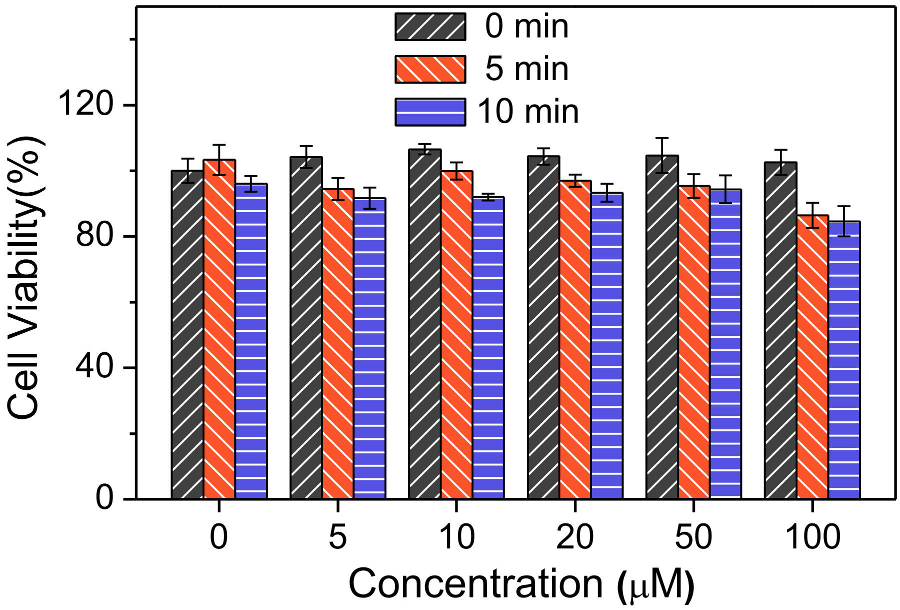

Fig. S3 Relative cell viability of HeLa cells incubated with PPE at a series of concentrations (0, 5, 10, 20, 50, and 100 μM) for 2 h without or with white light (400-800 nm, power: 1 W, 100 mW cm^{-2}) irradiation for different durations (5 and 10 min).

References

1. Z. Chen, P. Wu, R. Cong, N. Xu, Y. Tan, C. Tan and Y. Jiang, *ACS Appl. Mater. Interfaces*, 2016, **8**, 3567-3574.
